# Secoisopimaranes from *Salvia elegans* Vahl leaves as antibacterial agents against *Staphylococcus aureus*

**DOI:** 10.1038/s41598-025-19109-0

**Published:** 2025-10-08

**Authors:** Gabin Thierry M. Bitchagno, Erin M. Garcia, Sohini S. Bhatia, Scott Bintrim, Paula Coates, Deborah Mulligan, Monique S. J. Simmonds

**Affiliations:** 1 Royal Botanic Gardens Kew, Richmond, London TW9 3AE UK; 2 The Procter & Gamble Company, Mason Business Center, Mason, OH USA; 3 The Procter & Gamble Company, 452 Basingstoke Rd, Reading, RG2 0RX UK

**Keywords:** Metabolomic, *Salvia*, Lamiaceae, Secoisopimarane, *Staphylococcus aureus*, Structure elucidation, Biochemistry, Chemistry

## Abstract

The genus *Salvia* contains around 1000 species and is primarily known to produce caffeic acid polymers and diterpenes. Of the bi- and tricyclic classes of diterpenes found in *Salvia*, isopimaranes are the least represented. The bio-guided metabolomic analysis of the leaves of *Salvia elegans* Vahl revealed the occurrences of three secoisopimaranes (**1**–**3**), of which the known 3,4-secoisopimara-7,15-dien-3-oic acid was the most abundant and showed potent antimicrobial activity against *Staphylococcus aureus* with a MIC = 15.6 *µ*g/mL (51.6 *µ*M). All three diterpenes were detected in domesticated and wild specimens of the closely related species *Salvia cinnabarina* M.Martens & Galeotti, but not in other species reportedly to be closely related to *S. elegans*. Diterpenoids from *Salvia* are an interesting group of biologically active molecules and their distribution within the genus justifies further study.

**Supplementary Information:**

The online version contains supplementary material available at 10.1038/s41598-025-19109-0.

## Introduction

The pineapple sage (*Salvia elegans* Vahl) is a hardy shrub that originates from Mexico and other Central American countries like Guatemala and Honduras. Locally its leaves and flowers are used to make drinks and salads, and it is used in traditional remedies to relieve anxiety and insomnia^1^. In a recent study, the triterpene ursolic acid and flavonoid 5-*O*-(6-rhamnosylglucoside)−7-hydroxy-4’-methoxyflavanone were the main active principles linked to the species anxiolytic property^2^. Overall, species of *Salvia* are primarily known to produce caffeic acid, polyphenolic compounds such as rosmarinic and salvianolic acids, and diterpenes^3^.

*Salvia* diterpenes are represented by bi- and tricyclic classes including abietanes, (iso)pimaranes, clerodanes and labdanes^3,4^. The structures of labdanes usually feature a fourth ring (usually derived from furane) from the oxidation of the compound side chain, whereas the C-ring of abietanes are commonly overoxidized to royleanones, a abietane-type characteristic of species of *Coleus*^4^. Some abietanes from species of *Salvia* have their ring A or B of the perhydrophenanthrene system sectioned leading to secoabietanes^3,4^. The less common group of diterpenes in species of *Salvia* is (iso)pimaranes of which only one secoisopimarane have been reported so far and that was from the leaves of *S. cinnabarina* M.Martens & Galeotti and featuring a 3,4-section of the diterpene ring^4,5^.

Morphologically, *S. elegans* is similar to *S. cinnabarina* as both plants exhibit similar foliage and flowers of the same shape and color. However, *S. cinnabarina* grows taller and produces a greater number of flowers than *S. elegans*. A chloroplast DNA study of species of *Salvia* within the *Calosphace* subgenus, where *S. cinnabarina* and *S. elegans* belong, shows a strong molecular relationship between the two species^6^. Therefore, it could be hypothesized that *S. elegans* could contain similar diterpenoid groups to *S. cinnabarina* such as the secoisopimarane.

This study was undertaken to see if this was in fact the case and to evaluate whether secoisopimaranes like some of the other diterpenes have activity against Gram-positive bacteria like *Staphylococcus aureus*. It is suggested that diterpenes in general disrupt microbial cell walls of Gram-positive bacteria while remaining ineffective on the membranes of Gram-negative bacteria. However, the specific chemical characteristics shared among diterpenes that account for this activity remain unclear. The study involved plants from the living and herbarium collections at the Royal Botanic Gardens, Kew.

## Results and discussion

**Fig. 1:**
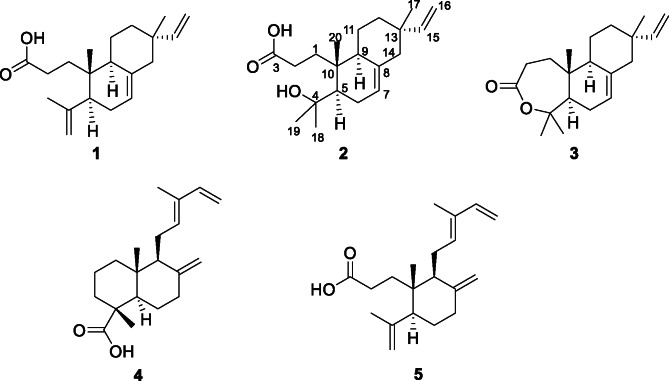
Structures of isolated compounds **1**–**5**.

*Salvia elegans* leaves were serially extracted in *n*-hexane, ethyl acetate (EtOAc) and 60% methanol. Extracts were tested for antimicrobial activity (both minimum inhibitory concentration (MIC) and minimum bactericidal concentration (MBC)) against *Escherichia coli*, *Staphylococcus aureus* and *Aspergillus brasiliensis*. None of the extracts tested against *E. coli* and *A. brasiliensis* exhibited activity, whereas the hexane extract inhibited *S. aureus* with a MIC of 62.5 *µ*g/mL (Table 1). Its fractionation with a flash chromatography system resulted in six fractions H1-H6, of which H2 and H3 exhibited moderate to strong inhibitory potential against *S. aureus* with MICs of 250 and 15.6 *µ*g/mL, respectively. The active fractions were examined following an NMR-based metabolomic approach described previously^7^. Fraction H3 (Fig. S2) was mainly composed of 3,4-secoisopimara-7,15-dien-3-oic acid (**1**) (Fig. 1 and S3) while the ^1^H NMR spectrum (Fig. S4) of H2 evidenced signals of a fatty acid. Therefore, compound **1** was purified during an untargeted analysis of both active fractions H2 and H3 on a HPLC system alongside two additional minor components, the secoisopimaranes (**2**–**3**) and labdane derivatives (**4**–**5**) (Fig. 1). Their structures were established by means of extensive NMR and MS analysis.

**Table 1:** Results of the antibacterial activity (*µ*g/mL) of extracts from *S. elegans*, fractions (H1-H6) and isolated compounds **1**–**3** against *Staphylococcus aureus*.

	Extracts			Fractions						Isolated compound		References	
	Hex	EtOAc	60%MeOH	H2	H3	H4	H4	H5	H6	1		Piroctone olamine	
MIC	62.5	500	1000	250	15.6	> 1000	> 1000	> 1000	> 1000	15.6		15.6	
MBC	500	> 1000	> 1000	> 1000	62.5	> 1000	> 1000	> 1000	> 1000	62.5		250	
R	8	–	–	–	4	–	–	–	–	4		16	

*R* = MBC/MIC, *Hex* EtOAc and 60%MeOH = Serial extracts in hexane, ethyl acetate and 60% MeOH in water; H1-H6 = fractions collected from the flash chromatography; **1** = 3,4-secoisopimara-7,15-dien-3-oic acid; **2** = *rel*-4-hydroxy-3,4-secoisopimara-7,15-dien-3-oic acid; **3** = *rel*-3,4-secoisopimara-7,15-dien-3,4-olide.

### Structure determination of new compounds

**Fig. 2:**
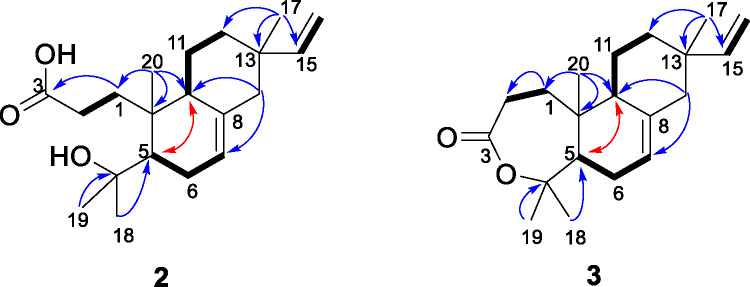
Selected COSY (bold lines), HMBC (blue arrows) and NOESY (red double arrows) correlations in compounds **2** and **3**.

Compound **2** was a colorless oil. Its negative-mode HRESIMS exhibited a deprotonated molecular ion peak [M-H] ^−^ at *m/z* 319.2275 for the molecular formula C_20_H_31_O_3_^−^ (calcd for C_20_H_31_O_3_^−^, *m/z* 319.2279). Its NMR spectra (Fig. S7-S12) provided evidence of a vinyl group at *δ*_H_ 5.82 (dd, *J* = 10.6, 17.7 Hz, H-15)/*δ*_C_ 150.1 (C-15), 4.90 (dd, *J* = 1.3, 10.6 Hz, H-16a) and 4.95 (dd, *J* = 1.3, 17.7 Hz, H-16b)/*δ*_C_ 109.5 (C-16) along with a broad doublet of olefin at *δ*_H_ 5.34 (*J* = 3.5 Hz, H-7)/*δ*_C_ 121.1 (C-7) and four angular methyls at *δ*_H_ 0.89 (s, H-17)/*δ*_C_ 21.4 (C-17), 1.03 (s, H-20)/*δ*_C_ 17.9 (C-20), 1.29 (s, H-19)/*δ*_C_ 34.4 (C-19) and 1.40 (s, H-18)/*δ*_C_ 26.5 (C-18). As it stands, one might think that these characteristics are those of the diterpene isopimaric acid, which was isolated from *Aeollanthus buchnerianus*^7^. However, the methyls H-18 and H-19 were constitutive of a 2-hydroxyisopropyl unit, supported by the HMBC cross peaks (Fig. 2) from one another as well as from the correlations either methyls exhibited to a methine at *δ*_C_ 49.3 (C-5) and an oxygenated carbon at *δ*_C_ 76.3 (C-4). This suggested an opened A-ring in compound **2** unlike isopimaric acid. Indeed, the methyl H-20 also showed correlations to the methine C-5, as expected in the A-ring of terpene skeletons, a second methine at *δ*_C_ 44.2 (C-9) and a methylene at *δ*_C_ 32.3 (C-1). The latter, with protons resonating at *δ*_H_ 1.84/2.28, was further engaged in a spin coupling with a second methylene at *δ*_H_ 2.28/2.72 as evidenced by the^1^H-^1^H COSY spectrum (Fig. S9) of compound **2**. The HMBC spectrum also exhibited correlations from H-1 to C-2 (*δ*_C_ 29.7) and a carbonyl at *δ*_C_ 177.4 (C-3). Further evidence in the^1^H^1^, H COSY spectrum of compound **2** supported a spin system between the methine H-9 and the methylenes H-11 (*δ*_H_ 1.39/1.56) and H-12 (*δ*_H_ 1.39/1.52). Which, when combined to the fact that the HMBC spectrum (Fig. S11) exhibited correlations from the methyl H-17 to C-12 (*δ*_C_ 35.9), C-15, C-13 (*δ*_C_ 36.8) and C-14 (*δ*_C_ 45.9), confirmed the (iso)pimarane nature of the structure of **2**. The relative stereochemistry of compound **2** was similar to that of (iso)pimaranes where the methyl H-20 and the methine H-5 are antiperiplanar while H-5 and H-9 are *cis* as judged by the correlations evidenced in its NOESY spectrum between H-20 and H-18; H-5 and H-19 or H-5 and H-9. In addition, the remaining stereochemistry at C-13 was established as *S* as the methyl C-17 resonated at 21.4 ppm in accordance with previously detailed method^7,8^. Thus, compound **2** is a new derivative of secoisopimaranes and characterized *rel*−4-hydroxy-3,4-secoisopimara-7,15-dien-3-oic acid.

Likewise, the positive-mode HRESIMS of compound **3** exhibited a protonated molecular ion peak [M + H]^+^ at *m/z* 303.2318 for the molecular formula C_20_H_31_O_2_^+^ (calcd for C_20_H_31_O_2_^+^, *m/z* 303.2319). Its NMR spectra (Fig. S15-S20) exhibit some of the resonances captured above for compound **2**. Mainly, the vinyl group ABX system at *δ*_H_ 5.82 (dd, *J* = 10.8, 17.6 Hz, H-15)/*δ*_C_ 149.8 (C-15), 4.91 (dd, *J* = 1.2, 10.8 Hz, H-16) and 4.96 (dd, *J* = 1.2, 17.6 Hz, H-16)/*δ*_C_ 109.6 (C-16), the olefin at *δ*_H_ 5.41 (m, H-7)/*δ*_C_ 121.0 (C-7) and the angular methyls at *δ*_H_ 0.90 (s, H-17)/*δ*_C_ 21.5 (C-17), 1.06 (s, H-20)/*δ*_C_ 15.3 (C-20), 1.45 (s, H-19)/*δ*_C_ 34.0 (C-19) and 1.58 (s, H-18)/*δ*_C_ 24.2 (C-18) (Table 2). Similarly, the HMBC correlations (Fig. 2) of all methyl groups support a isopimarane skeleton for compound **3** including a hydroxyisopropyl fragment at C-5 as for compound **2**, but with slight differences. Indeed, the carbonyl C-3 (*δ*_C_ 175.8) was shielded by 1.8 ppm while C-4 (*δ*_C_ 86.3) was deshielded by 10 ppm compared to the same carbon atoms in compound **2**. Surprisingly, compound **3** was not ionizable in negative mode but in positive mode which indicates that its structure lacks the acid or hydroxyl functions that have made compound **2** active in both modes. Thus, the structure of **3** was proposed with a lactone between C-3 and C-4. This ring closure can also justify the shielding of H-20 and C-18 or the deshielding of C-5, H-18 and H-19. The relative stereochemistry of compound **3** is similar to that of compound **2** where the methyl H-20 and the methine H-5 are periplanar while H-5 and H-9 are *cis* and the configuration around C-13 is *S* as its NOESY spectrum provided the same evidence as with compound **2** including correlations between H-20 and H-18 as well as between H-5 and H-19. Compound **3** is a new derivative of compound **2** and characterized as *rel*−3,4-secoisopimara-7,15-dien-3,4-olide.

**Table 2:** ^[1]^H (400 MHz) and ^13^C (100 MHz) NMR spectroscopic data for compounds **2** and **3** (*δ* in ppm).

Position	2		3	
1	1.84, m 2.28, m	32.3, CH_2_	2.70, dd (2.2, 8.7) 2.74, dd (2.2, 10.6)	33.2, CH_2_
2	2.28, m 2.72, t (12.4)	29.7, CH_2_	1.6, m 2.00, m	31.8, CH_2_
3		177.4, C		175.8, C
4		76.3, C		86.3, C
5	1.74, dd (4.5, 11.3)	49.3, CH	1.83, dd (4.1, 11.6)	51.5, CH
6	2.00, m	27.2, CH_2_	2.03, m	26.9, CH_2_
7	5.34, br d (3.5)	121.1, CH	5.41, m	121.0, CH
8		135.9, C		135.7, C
9	1.88, m	44.2, CH	1.81, m	49.2, CH
10		38.9, C		38.4, C
11	1.56, m 1.39, m	19.3, CH_2_	1.39, m 1.63, m	20.2, CH_2_
12	1.39, m 1.52, m	35.9, CH_2_	1.39, m 1.52, m	36.0, CH_2_
13		36.8, C		36.8, C
14	1.99, m	45.9, CH_2_	1.99, m	46.0, CH_2_
15	5.82, dd (10.6, 17.7)	150.1, CH	5.82, dd (10.8, 17.6)	149.8, CH
16	4.90, dd (1.3, 10.6) 4.95, dd (1.3, 17.7)	109.5, CH_2_	4.91, dd (1.2, 10.8) 4.96, dd (1.2, 17.6)	109.6, CH_2_
17	0.89, s	21.4, CH_3_	0.90, s	21.5, CH_3_
18	1.40, s*	26.5, CH_3_*	1.58, s*	24.2, CH_3_*
19	1.29, s*	34.4, CH_3_*	1.45, s*	34.0, CH_3_*
20	1.03, s	17.9, CH_3_	1.06, s	15.3, CH_3_

*Positions are interchangeable.

**Fig. 3:**
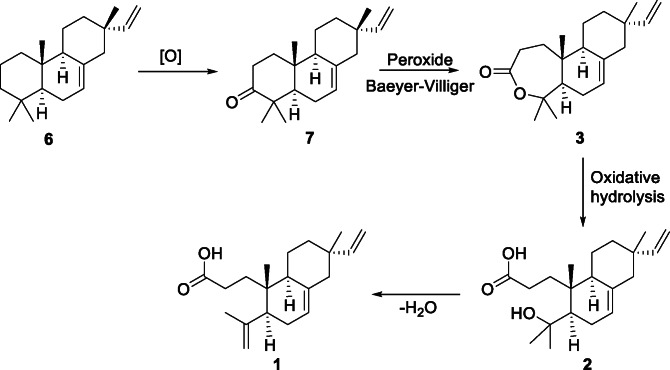
Proposed biosynthesis route to compounds **1**–**3**.

Considering the significant yield of the plant material in the secoisopimarane **1** as compared to either compounds **2** or **3**, one can consider the leaves of *S. elegans* as the biofactory of compound **1**. Its biosynthesis is proposed to have begun with the oxidation of isopimara-7,15-diene (**6**) to the known isopimara-7,15-dien-3-one (**7**) which then undergoes a Baeyer-Villiger oxidation to compound **3**. The oxidative hydrolysis of **3** followed and leads to compound **2** which continues following an elimination (dehydration) reaction to compound **1** (Fig. 3). This probable protocol can be achieved by either enzymatic or chemical routes.

### Antibacterial assay results

Compound **1** was the most abundant compound of either fraction H3 or extract accounting for 0.11 mg/g of the dried materials. It was also the only compound tested for potency against *S. aureus* as the other compounds were isolated in very low yields. Compound **1** replicates the activity of the active fraction H3 with a MIC of 15.6 *µ*g/mL (51.6 *µ*M) similar to the activity of the positive reference, piroctone olamine, (MIC 15.6 *µ*g/mL or 52.3 *µ*M). In addition, compound **1** was bactericidal while the hexane extract was bacteriostatic. This is the first report on the antibacterial activity of the hexane extract of *S. elegans* and the secoisopimarane **1**. None of the other more polar extracts showed activity against *S. aureus* and none of the samples were active against the other microbes tested^9^.

Surprisingly, this is only the second report of secoisopimaranes in *Salvia*, following the work of Romussi et al.^5^ which first highlighted the occurrence of compound **1** in *S. cinnabarina*. Secoisopimaranes have been mainly reported in species of *Isodon* and *Orthosiphon*, two closely related genera to *Salvia*, where the preference for secoisopimaranes have always been for the 3,4-seco type^10–14^ as opposed to the 9,10-seco type found exclusively in *Kaempferia sp*^15–17^.

### Distribution of the isopimaranes across salvia species

If secoisopimarane **1** is to be utilized as an antibacterial compound then it is important to know what other species of *Salvia* produces it and at what yields. The genus *Salvia* contains around 1000 species grouped in ten subgenus made of various clades^18^. For instance, *S. elegans* is consistently placed in the *Calosphace* subgenus but in different clades^6,18^. In the general classification of the genus, *S. elegans* forms a clade (Fig. S21A) with *S. muelleri* Epling, *S. lycioides* A. Gray, *S. coahuilensis* Fernald, *S. chamaedryoides* Cav. *S. microphylla* Kunth, *S. darcyi* J.Compton, *S. karwinskii* Benth., *S. curviflora* Benth. and *S. wagneriana* Pol^18^. In contrast, a clade within the classification of the *Calosphace* subgenus relates *S. elegans* to *S. cinnabarina*, *S. clinopodioides* Kunth, *S. ramosa* Brandegee and *S. regla* Cav. (Fig. S21B)^6^. Based on the position of *S. elegans* in these papers, leaves from 41 specimens (Table 3) were sampled from the Herbarium at RBG Kew. The sampling included 2–5 wild specimens per species and 1–2 specimens of the same species cultivated in Europe. Collected materials were ground separately into powder and extracted in *n*-hexane. After removal of the solvent on a centrifugal evaporator, the extracts were dissolved in a known amount of acetonitrile (ACN) and injected into the Orbitrap Exploris 120 LC-MS along with the same solution from dried plant materials of *S. elegans* collected from the Living Collection at RBG Kew. Generated LC-MS data were processed on MS Dial and aligned referenced to the pool sample data. The peak areas of detected ions in each sample were extracted and only the corresponding features of compounds **1**–**3** were retained for comparison.

**Table 3:** Specimen details collected from the herbarium at RBG kew.

Barcode	Genus	Species	Authority	Collector(s)	Date of Collection	Country	Category
K004935165	*Salvia*	*elegans*	Vahl	Nahu Gonzales Castaneda	17.02.2009	Mexico	Wild
K000967561	*Salvia*	*elegans*	Vahl	R. Bye and E. Linares	27.01.1982	Mexico	Wild
K000735660	*Salvia*	*elegans*	Vahl	M Sparrow and P. Brewster	5.11.1993	Mexico	Wild
K000966617	*Salvia*	*elegans*	Vahl	MF Gardner and SG Knees	22.10.1992	Mexico	Wild
K000966619	*Salvia*	*elegans*	Vahl	St Pierre	22.3.1928	Mexico	Wild
K004938973	*Salvia*	*darcyi*	J.Compton	EP Santos	24.04.2003	France	Cultivated
K000568291	*Salvia*	*darcyi*	J.Compton	Campton	20.7.2011	Kew, UK	Cultivated
K004938931	*Salvia*	*elegans var. Sonoransis*	Vahl	J Marnier	26.10.1964	France	Cultivated
K004938927	*Salvia*	*elegans*	Vahl		20.8.1976	Kew, UK	Cultivated
K000248130	*Salvia*	*darcyi*	J.Compton	MR Garcia Pena	20.10.1991	Coll. Sierra Madre Oriental	Wild
K004935757	*Salvia*	*columbariae*	Benth.	Vargas, Armando Ponce	31.5.2013	Mexico	Wild
K000926479	*Salvia*	*columbariae*	Benth.	H. Van der Werff	4.1976	Mexico	Wild
K004935613	*Salvia*	*karwinskii*	Benth.	R. Cqrbqllo	30.11.2004	El Salvador, Mexico	Wild
K004935612	*Salvia*	*kawinskii*	Benth.	D. Rodriguez	21.1.2016	EL Salvador, Mexico	Wild
K004935605	*Salvia*	*kawinskii*	Benth.	Randy Evans	16.2.1993	Honduras	Wild
K004935639	*Salvia*	*wagneriana*	Pol.	JC Sandino	21.2.1980	Nicaragua	Wild
K004935649	*Salvia*	*wagneriana*	Pol.	Carl Epling	1861	Guatemala	Wild
K004935653	*Salvia*	*wagneriana*	Pol.	E. Matuda	2.4.1939	Mexico	Wild
K000967728	*Salvia*	*coahuilensis*	Fernald	CG Pringle	16.8.1901	Mexico	Wild
K004935184	*Salvia*	*lycioides*	A.Gray	K. Prestson Mafham	1978	Mexico	Wild
K000967717	*Salvia*	*chamaedryoides*	Cav.	CC Parry	4.1999	Mexico	Wild
K000967713	*Salvia*	*chamaedryoides*	Cav.	CG Pringle	18.9.1902	Mexico	Wild
K000288114	*Salvia*	*curviflora*	Benth.	Carl Epling	1928	Mexico	Wild
K004935465	*Salvia*	*microphylla var. Wislizeni*	A.Gray	A Paton	22.1.1993	Mexico	Wild
K004935467	*Salvia*	*microphylla var. neurepia*	(Fernald) Epling	E BOURGEAU	1865	Mexico	Wild
KOO4939215	*Salvia*	*microphylla*	Benth.	Bramley GLC	23.7.2007	Kew, UK	Cultivated
K004939208	*Salvia*	*microphylla var. neurepia*	(Fernald) Epling	J. Marnior	26.10.64	France	Cultivated
K004938918	*Salvia*	*chamaedryoides*	Cav.	A. Bisio	10.2.2008	Italy	Cultivated
K004938885	*Salvia*	*columbariae*	Benth.	RM Harley	27.6.1969	Kew, UK	Cultivated
K004938873	*Salvia*	*curviflora*	Benth.	G Bramley	2.10.2010	Kew, UK	Cultivated
K004939605	*Salvia*	*wagneriana*	Pol.	Lord Jalbot Malakide	3.2.1965	Kew, UK	Cultivated
K004935485	*Salvia*	*regla*	Cav.	GB Hinton	28.8.1991	Mexico	wild
K004935482	*Salvia*	*regla*	Cav.	Katty Peterson	1.10.1982	Mexico	wild
K000966612	*Salvia*	*cinnabarina*	M.Martens & Galeotti	JPM Brenan	26.10.1977	Mexico	Wild
K000966606	*Salvia*	*cinnabarina*	M.Martens & Galeotti	Thomas B Croat	19.1.1979	Mexico	wild
K000966597	*Salvia*	*cinnabarina*	M.Martens & Galeotti	Thomas B Croat	23.1.1987	Guatemala	wild
K004935164	*Salvia*	*cinnabarina*	M.Martens & Galeotti	Randy Evans	9.2.1993	Hunduras	wild
K000265848	*Salvia*	*clinopodioides*	Kunth	Esteban Martinez S	13.10.1983	Mexico	wild
K000266711	*Salvia*	*clinopodioides*	Kunth	James L reveal	26.9.1973	Mexico	wild
K004938907	*Salvia*	*cinnabarina*	M.Martens & Galeotti	A. Bisio	10.02.2008	Italy	Cultivated
K004939391	*Salvia*	*regla*	Cav.	S. Walker	17.10.1983	USA	Cultivated

The results (Fig. S22) show that the three secoisopimaranes are only detected in various specimens of *S. cinnabarina* and *S. elegans*. Both wild and cultivated materials showed relative levels of the target compounds. The three compounds were more abundant in specimens of *S. cinnabarina* than *S. elegans*. Therefore, the former could be a better source of the three compounds. The absence of compounds **1**–**3** in the other species does not enforce any particular phylogeny, although it does support the molecular phylogeny of Lara-Cabrera et al.^6^ that places both these species together. The lack of the secoisopimaranes **1**–**3** in other species could be an indication to consider them for authentication of both species. This could be assessed by extending the qualitative analysis to other species and subgenus of *Salvia*. Such an assessment would provide additional insights to solve the still opened question of the distribution of (iso)pimaranes in *Salvia*.

## Methods

### General experimental procedure

LC–MS grade solvents (acetonitrile, methanol) and formic acid were obtained from Fisher Scientific (Loughborough, UK) and milliQ water was used for HPLC and LC-MS analysis. NMR spectra were acquired on a Bruker Avance-III ^1^H NMR: 400 MHz and ^13^C NMR: 100.1 MHz) spectrometer equipped with a 5 mm cryoprobe. Chemical shifts were referenced to residual solvent signals and reported in parts per million (ppm). Spectra were processed using Bruker NMR academic Topspin software. Mass spectra were collected on a Orbitrap Exploris mass spectrometer, equipped with an Orbitrap Exploris 120 with a heated ESI source (Thermo Scientific, Germany), acquired in both negative and positive modes with a resolution of 60,000 over *m/z* 125–1800 under various acquisition parameters Like source voltages, sheath gas, auxiliary gas, sweep gas and capillary temperature set to 2.5 kV (negative mode) and 3.5 kV (positive mode), 50 (arbitrary units), 10 (arbitrary units), 1 (arbitrary units) and 350 °C, respectively. Automatic MS–MS fragmentation was performed on top four ions of the TIC using an isolation width of *m/z* 2. High-energy C-trap Dissociation with a normalized collision energy of 40 and an activation time of 0.1 ms was served to fragment ions. The MS unit is interfaced with a Vanquish Core UHPLC system, which includes a Vanquish diode array detector (VH-D10) operating at four wavelengths: 210 nm, 254 nm, 300 nm, and 366 nm. Samples are injected using an autosampler maintained at 30 °C, while the analytical column is kept at a constant temperature of 35 °C. The injection volume for each sample is 1 *µ*L with a gradient of acetonitrile (B) in water (A) (0–5 min, 10% B; 5–20 min, 10 to 100% B, 20 to 30 min, 100% B and 30 to 35 min, 10% B). Collected data were inspected using Xcalibur v. 4.2.47 (Thermo Fisher Scientific). Chemical profiling of extracts was conducted on a Biotage^®^ Isolera One system for splitting extracts into small fractions and a Waters Alliance 2695 HPLC system for isolation of compounds. A reversed-phase Discovery HS C-18 column (5 *µ*m, 10 mm × 250 mm i.d., Supelco, UK) maintained at 35 °C served in compounds isolation and purification over gradient of acetonitrile + 0.1% formic acid (A) and water (B).

### Plant material

The aerial parts of *Salvia elegans* were collected from the Living collection at RBG Kew. The specimen was fully verified by Dr Sven Landrein at RBG Kew where it is cultivated under the accession No 1994–2122. The material was freeze-dried, milled to a fine powder, and kept in the dark before being used.

### Extraction and purification of compounds

The plant powder (500 g) was serial extracted in *n*-hexane, EtOAc and 60% MeOH affording dried extracts of 1.14 g, 1.86 g and 2.56 g, respectively. The hexane extract was split into six fractions (H1-H6) following flash chromatography on a semi-prep Biotage Isolera system. The gradient was a stepwise increase of isopropanol (B) in MeOH (A) (3CV, 5% B; 3CV, 10% B, 3CV, 20% B, 3CV, 30% B; 3CV, 40% B, 3CV, 50% B and 3CV, 100% B), flowrate 30 mL/min, on a SNAP Ultra C18 60 g cartridge from Biotage. The fractions were dissolved in CDCl_3_ and submitted to^1^H NMR then to 2D NMR for chemical profiling. As a result of bio-guided protocol, only fractions H2 (30.9 mg) and H3 (224.3 mg) were further purified. Fraction H3 was dissolved in 4 mL of ACN + 10% DMSO and injected into the Waters system, eluting with a constant flow rate of 2 mL/min of a linear gradient of acetonitrile (D) in water (C) (0–5 min, 70% D; 5–60 min, 70 to 90% D, 60 to 70 min, 100% D and 70 to 75 min, 70% D). Compounds were detected at 210, 254, 300 and 354 nm and collected by time into glass tubes. Cumulative fractions from 32 injections of 100 *µ*L each were collected and dried using a GeneVac concentrator (Genevac, Suffolk, UK). Fraction H2 was not further purified. Collected fractions were dried and analyzed by NMR leading to compounds **1** (56.4 mg), **2** (3.1 mg), **3** (2.5 mg), **4** (< 1 mg), **5** (< 1 mg), oleanolic acid (4.2 mg) and ursolic acid (3.6 mg).

*rel-4-hydroxy-3*,*4-secoisopimara-7*,*15-dien-3-oic acid (****2****)*. Colorless oil; UV (MeOH) λ_max_ 218, 266 nm; (-)-HRESIMS, [M-H] ^−^ of *m/z* 319.2275 (calcd for C_20_H_31_O_3_^−^, 319.2279)^1^H and ^13^C NMR spectroscopic data are summarized in Table 2.

*rel-3*,*4-secoisopimara-7*,*15-dien-3*,*4-olide (****3****)*. Colorless oil; UV (MeOH) λ_max_ 218, 266 nm; (+)-HRESIMS, [M + H]^+^ of *m/z* 303.2318 (calcd for C_20_H_31_O_2_^+^, 303.2319);^1^H and ^13^C NMR spectroscopic data are summarized in Table 2.

### Qualitative and comparison analysis

A total of 41 specimens of species (aerial parts) closely related to *Salvia elegans* were collected from the Herbarium at RBG Kew. For each specimen, the milled plant material (15 mg) was suspended in 1.2 mL of *n*-hexane, vortexed vigorously for 10 s, heated in a water bath kept at 50 °C for 10 min and centrifuged at 15,600 rpm for 10 min. Part of the supernatant (1 mL) was transferred to a clean flask and dried using a centrifugal evaporator (Genevac, Suffolk, UK). These extracts were then dissolved in 1 mL ACN, sonicated and spined at 15,600 rpm for 10 min, 300 *µ*L of each extract was transferred into a glass autosampler vial for LC-MS analysis. The samples were all injected into the Orbitrap Exploris 120 using a gradient of ACN in H_2_O, the other parameters listed above remain unchanged. The LC-MS data generated were deconvoluted, aligned using a pool of all samples and extracted as peak areas using MS-Dial (https://systemsomicslab.github.io/compms/msdial/main.html). The processing of data was done using the mass tolerance, rt tolerance and minimum peak height of 0.01 Da, 0.04 min and 10^4^, respectively. The other parameters remained the same as default settings and no database was added for peak annotation. Both negative and positive modes were retrieved separately.

### Antimicrobial assays

Minimum Inhibitory Concentration (MIC) and Minimum Biocidal Concentration (MBC) assays were used to determine the minimum concentration of an active required to inhibit half of the growth of microorganisms. Samples were evaluated against a range of organisms in triplicate including the Gram-positive bacterium, *Staphylococcus aureus* ATCC6538, the Gram-negative strain, *Eschierichia coli* ATCC8739 and the mould *Aspergillus brasiliensis* ATCC16404. Microbial solutions were prepared in saline (bacteria) and saline with tween (mould) and adjusted turbidometrically to a target concentration of 10^7^ −10^8^ CFU/mL. This inoculum solution was further diluted in Tryptic Soy broth (*S. aureus* and *E. coli*) or Soya Dextrose broth (*A. brasiliensis*) to achieve a final inoculum level of approximately 10^5^ CFU/mL assay use. A stock 96-well plate of the extracts were prepared in dimethyl sulfoxide (DMSO) at a concentration of 20 mg/mL and serially diluted in DMSO. For each test plate, 5 *µ*L of each dilution (each well from the stock plate) was transferred to a new test plate and 195 *µ*L of inoculum in broth was added to each well. The *S. aureus* and *E. coli* plates were incubated on an orbital sharker for 18 ± 2 h at 32.5 °C. The *A. brasiliensis* plates were inoculated at 20–25 °C for 5 days. After incubation, the plates were visually assessed and the MICs were determined as the most dilute well with reduced growth (~ 50%) compared to a growth control. To determine the Minimum Biocidal Concentration (MBC), 10 *µ*L from each well was pipetted onto neutralizing agar (Modified Letheen Agar with Tween) and incubated for 24 h at 32.5 °C (*S. aureus* and *E. coli*) or 5 days at 20–25 °C for 5 days (*A. brasiliensis).* The MBC was determined as the most dilute concentration with no visible growth.

## Supplementary Information

Below is the link to the electronic supplementary material.

Supplementary Material 1

## Data Availability

The Supporting Information is available including 1D and 2D NMR spectra, HRMS spectra, and UV spectrum for compounds **2** and **3**. The NMR and LC–MS raw data generated and/or analysed during the current study are available from the corresponding author (Gabin Bitchagno) on request.
