# Supplementary material for: Secoisopimaranes from Salvia elegans Vahl leaves as antibacterial agents against Staphylococcus aureus
Source: Sci Rep. 2025 Oct 8;15:35200. doi: 10.1038/s41598-025-19109-0 (PMC12508438; doi:10.1038/s41598-025-19109-0)
Supplement: Supplementary file 1 — Supplementary Material 1 [file 41598_2025_19109_MOESM1_ESM.docx]

**Secoisopimaranes from *Salvia elegans* Vahl Leaves as Antibacterial Agents Against *Staphylococcus aureus***

Gabin Thierry M. Bitchagno^1^ *, Erin M. Garcia^2^, Sohini S. Bhatia^2^, Scott Bintrim^2^, Paula Coates^3^, Debbie Mulligan^2^, Monique S.J. Simmonds^1^ *

^1^Royal Botanic Gardens, Kew, Richmond, London, TW9 3AE

^2^The Procter & Gamble Company, Mason Business Center, Mason, Ohio, USA

^3^The Procter & Gamble Company, 452 Basingstoke Rd, Reading RG2 0RX

**Table of contents**

Figure S1. Base peak chromatograms ESI- (top), ESI+ (middle) and PDA (bottom) of the hexane extract of *S. elegans*.

Figure S2. 1H NMR and Base peak chromatograms ESI- (top), ESI+ (middle) and PDA (bottom) of fraction H3.

Figure S3. ^1^H NMR and HR-ESI-MS/MS of compound **1**.

Figure S4. ^1^H NMR and Base peak chromatograms ESI- (top), ESI+ (middle) and PDA (bottom) of fraction H2.

Figure S5. HRESI-MS/MS spectra of compound **2**

Figure S6. UV trace of compound **2**

Figure S7. ^1^H NMR spectrum (400 MHz) of compound **2** in CDCl_3_

Figure S8. ^13^C NMR spectrum (100 MHz) of compound **2** in CDCl_3_

Figure S9. ^1^H-^1^H COSY spectrum of compound **2** in CDCl_3_

Figure S10. HSQC spectrum of compound **2** in CDCl_3_

Figure S11. HMBC spectrum of compound **2** in CDCl_3_

Figure S12. NOESY spectrum of compound **2** in CDCl_3_

Figure S13. HRESI-MS/MS of compound **3**

Figure S14. UV trace of compound 3

Figure S15. ^1^H NMR spectrum (400 MHz) of compound **3** in CDCl_3_

Figure S16. ^13^C NMR spectrum (100 MHz) of compound **3** in CDCl_3_

Figure S17. ^1^H,^1^H COSY spectrum of compound **3** in CDCl_3_

Figure S18. HSQC spectrum of compound **3** in CDCl_3_

Figure S19. HMBC spectrum of compound **3** in CDCl_3_

Figure S20. NOESY spectrum of compound **3** in CDCl_3_

Figure S21. Related species of *S. elegans* as reported by Rose et al.[1] (A) and Lara-Cabrera et al.[2] (B)

clade

Figure S22. Distribution chart of compounds **1**-**3** across *S. elegans* clade

Figure S1. Base peak chromatograms ESI- (top), ESI+ (middle) and PDA (bottom) of the hexane extract of *S. elegans*.


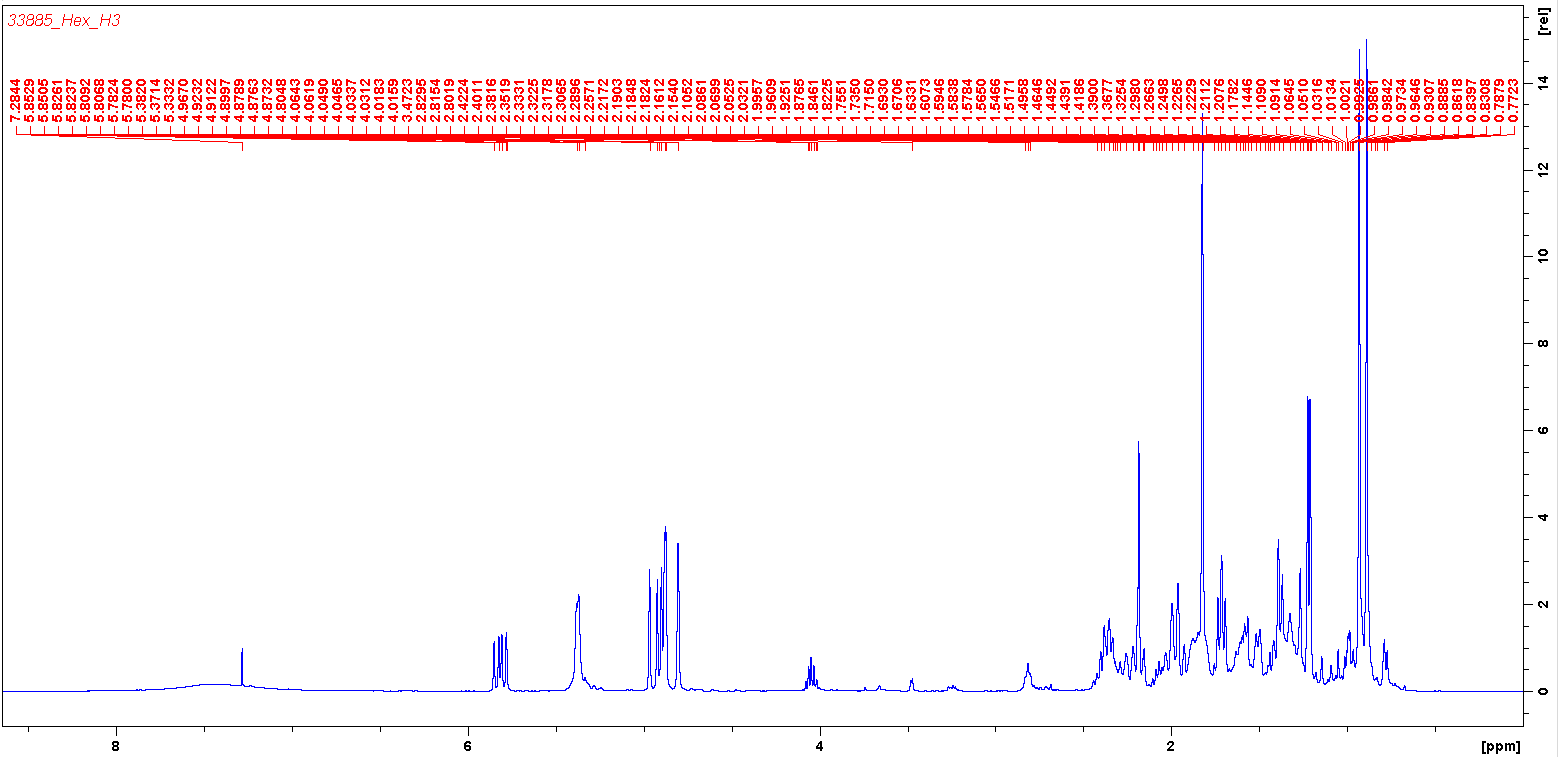


Figure S2. ^1^H NMR and Base peak chromatograms ESI- (top), ESI+ (middle) and PDA (bottom) of fraction H3.


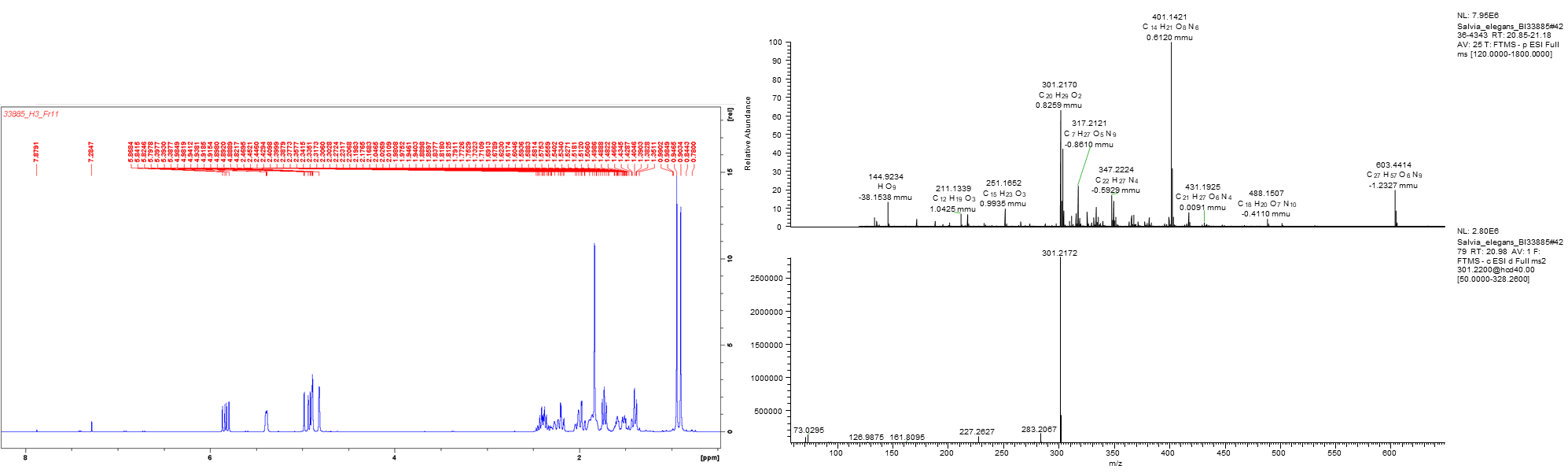


Figure S3. ^1^H NMR and HR-ESI-MS/MS of compound **1**.


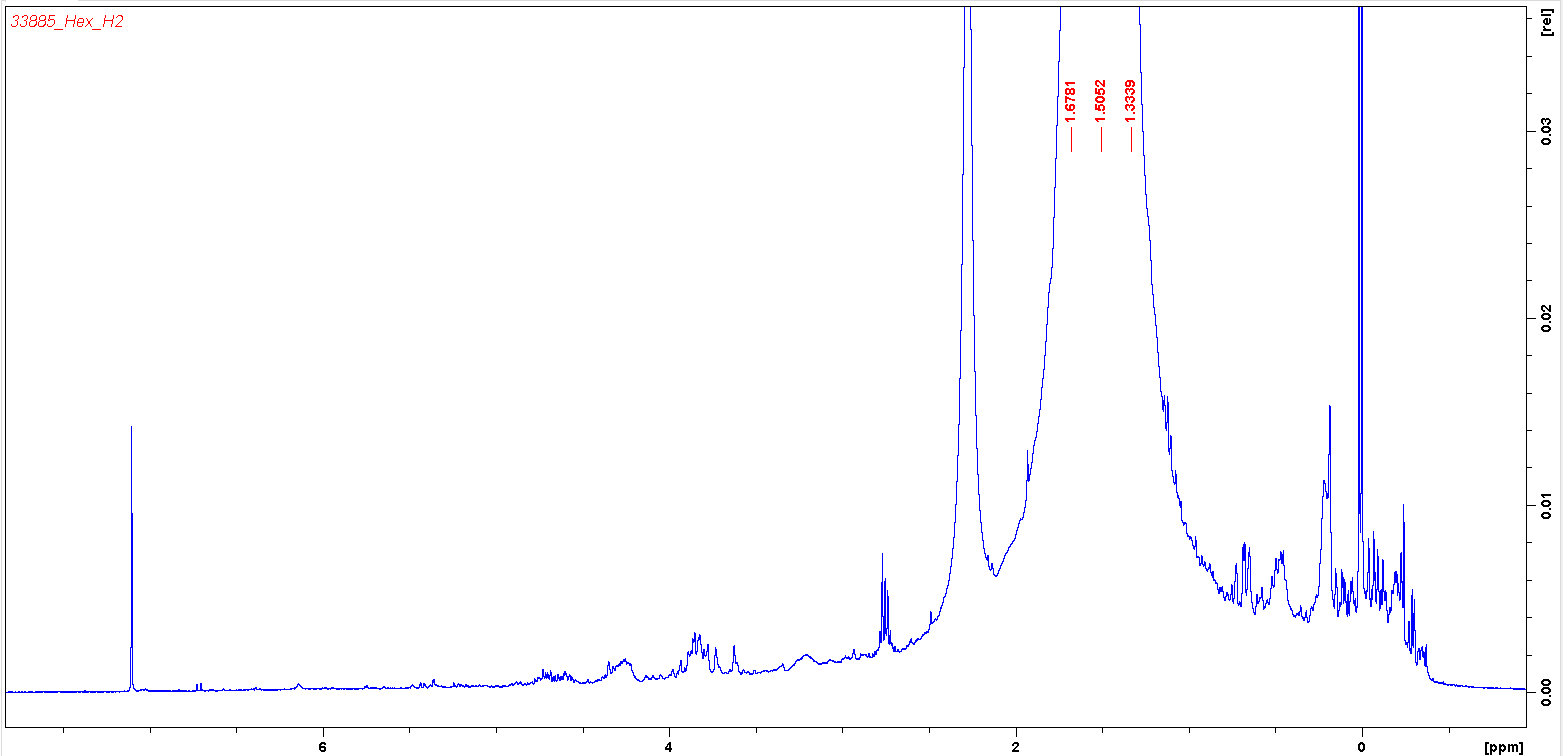


Figure S4. ^1^H NMR and Base peak chromatograms ESI- (top), ESI+ (middle) and PDA (bottom) of fraction H2.

Figure S5. HRESI-MS/MS spectra of compound **2**

Figure S6. UV trace of compound **2**


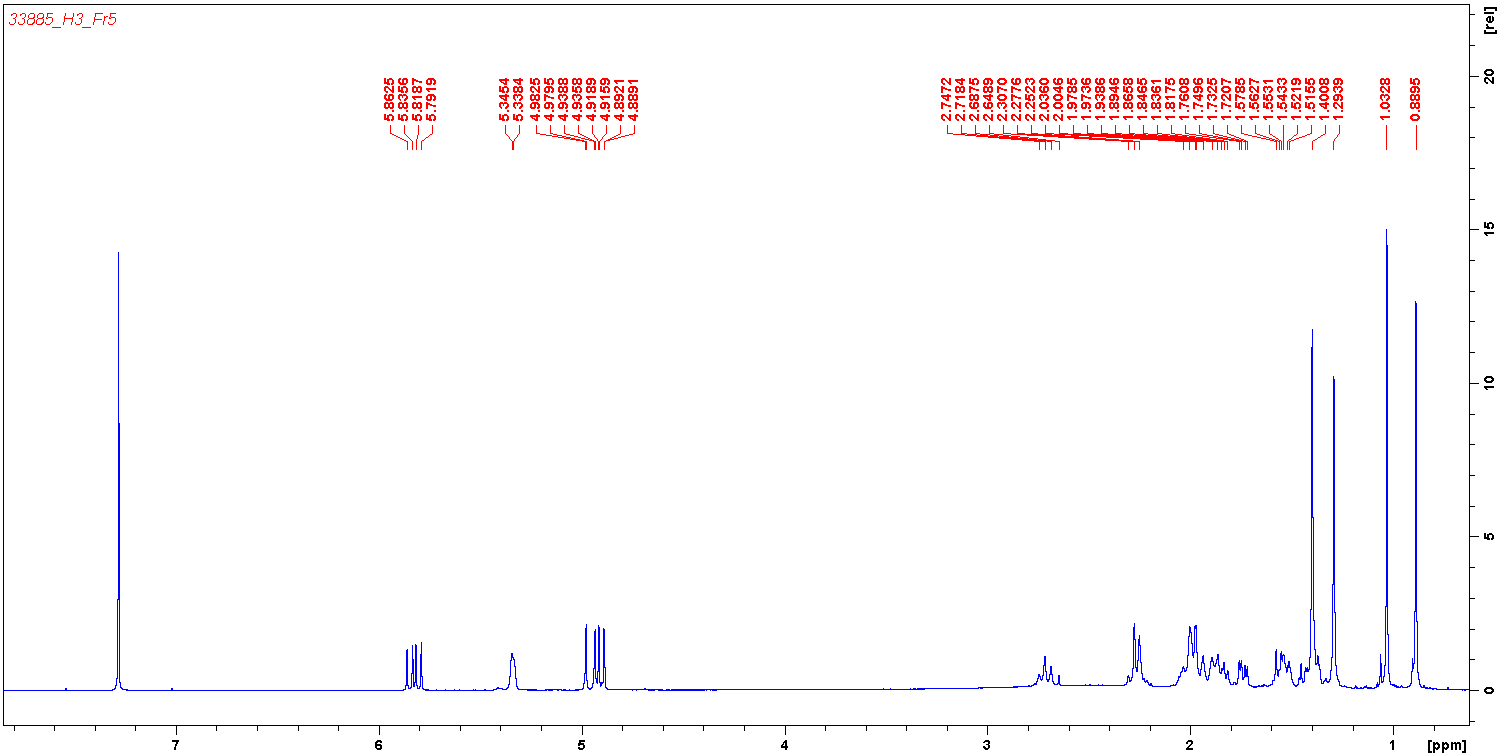


Figure S7. ^1^H NMR spectrum (400 MHz) of compound **2** in CDCl_3_


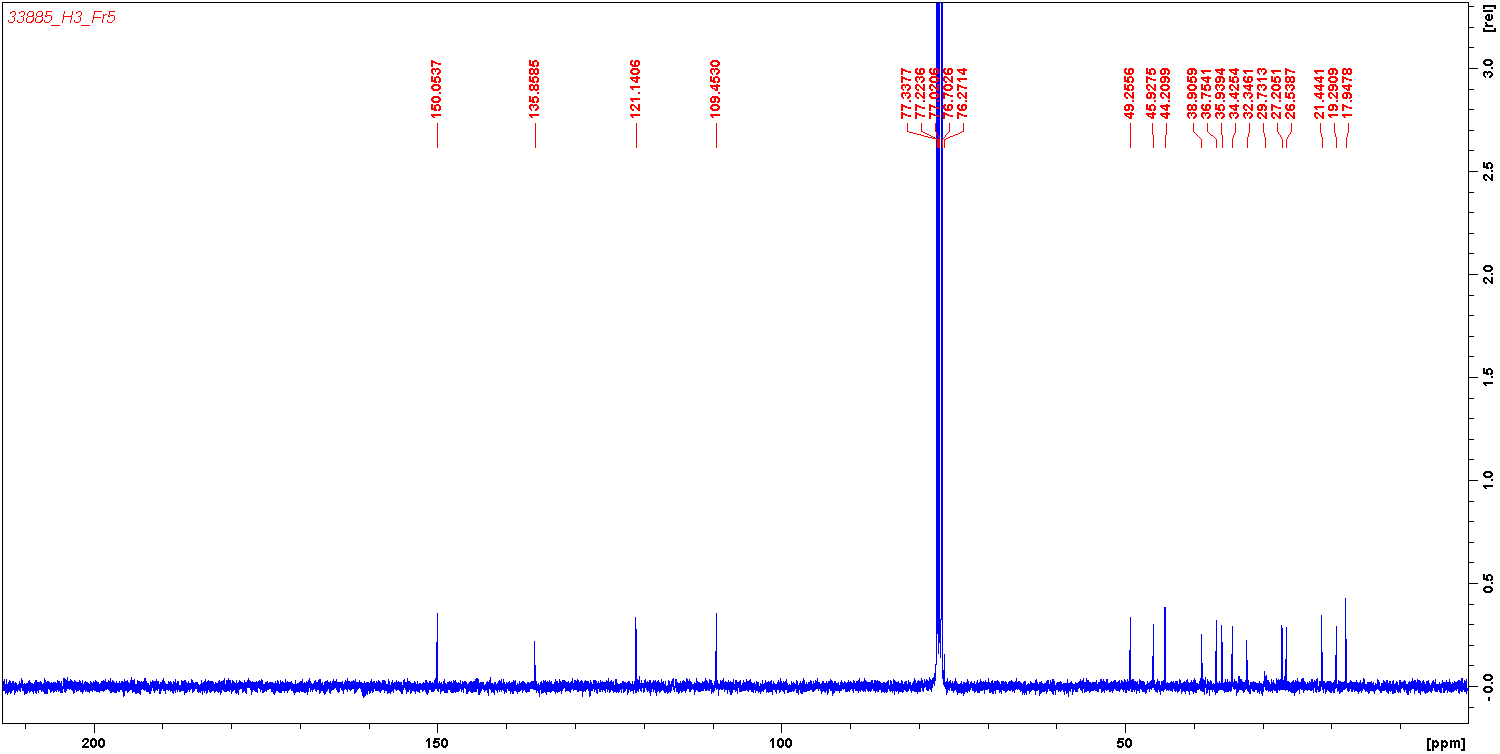


Figure S8. ^13^C NMR spectrum (100 MHz) of compound **2** in CDCl_3_


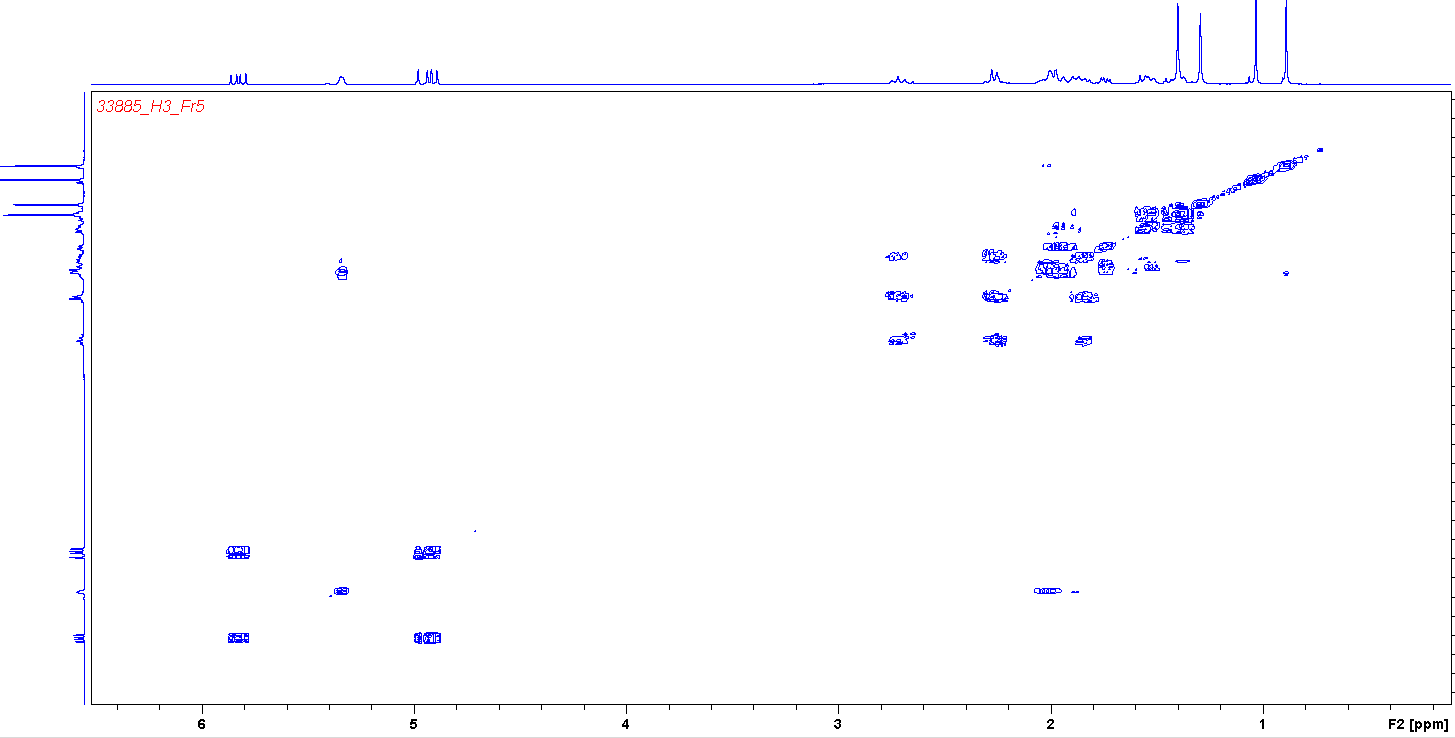


Figure S9. ^1^H-^1^H COSY spectrum of compound **2** in CDCl_3_


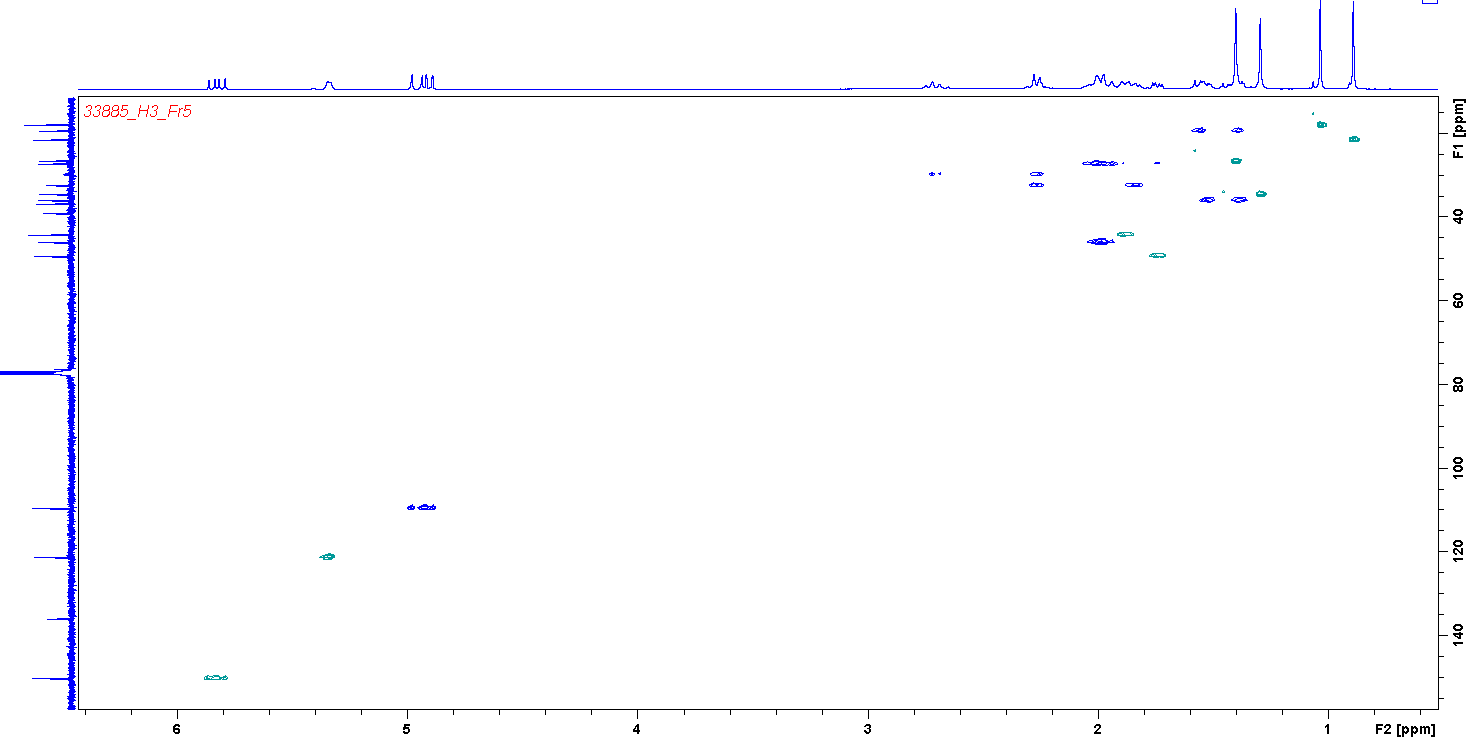


Figure S10. HSQC spectrum of compound **2** in CDCl_3_


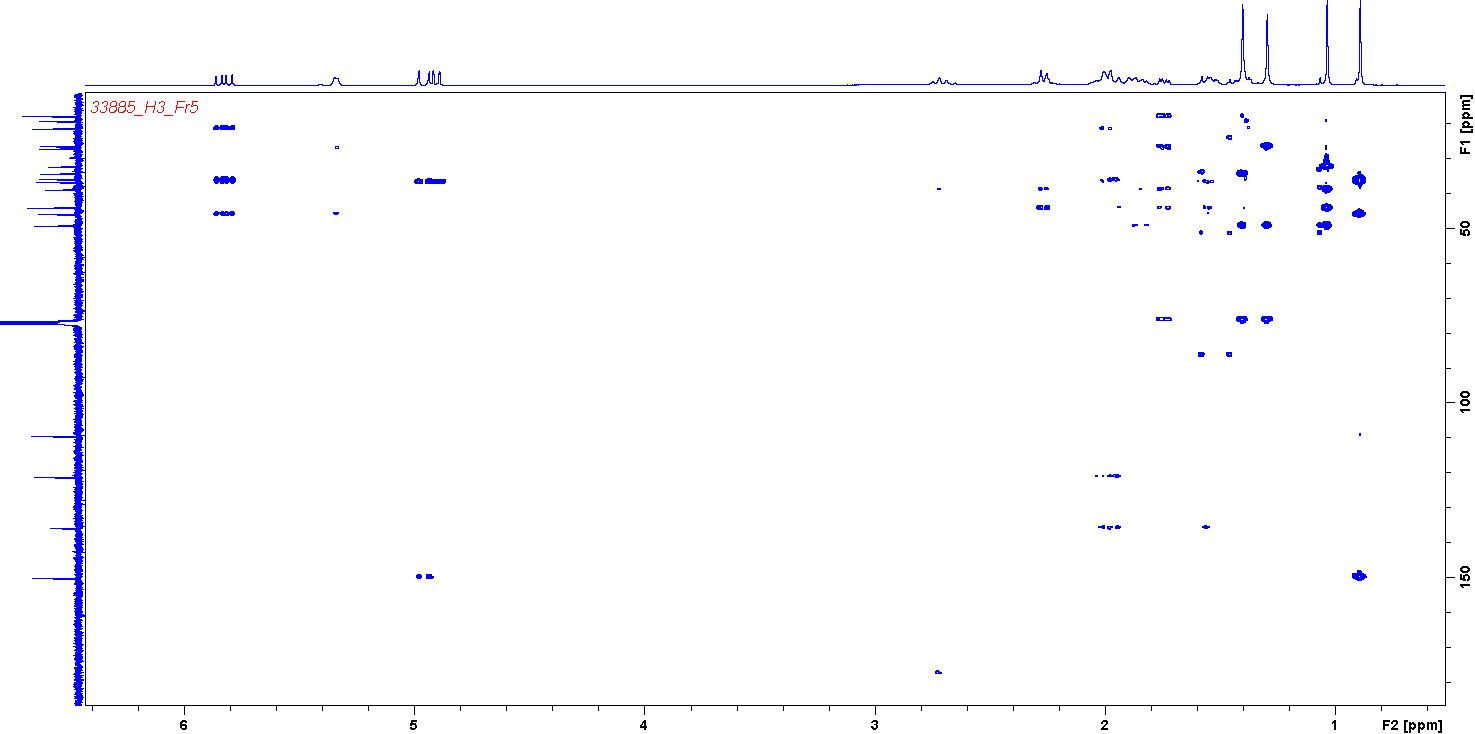


Figure S11. HMBC spectrum of compound **2** in CDCl_3_


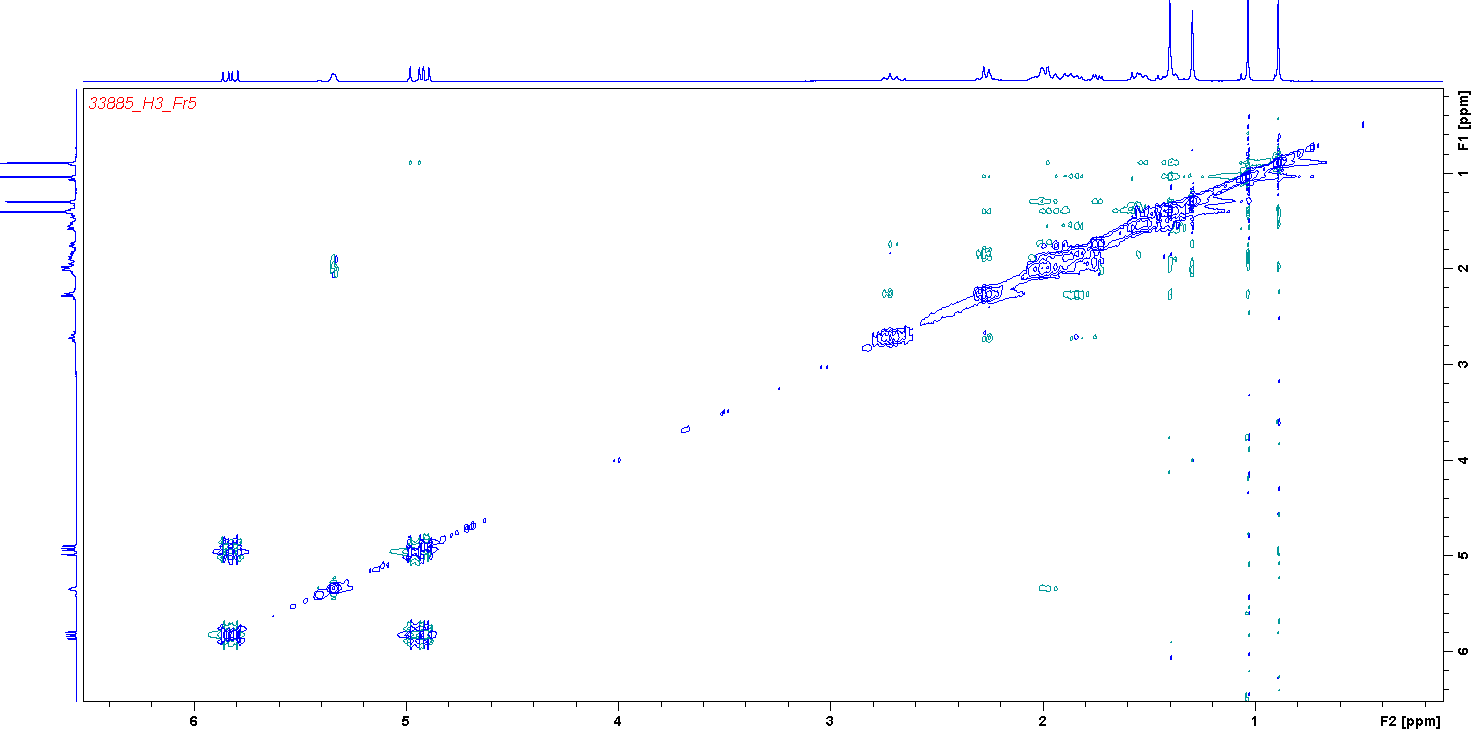


Figure S12. NOESY spectrum of compound **2** in CDCl_3_

__

Figure S13. HRESI-MS/MS of compound **3**

Figure S14. UV trace of compound 3


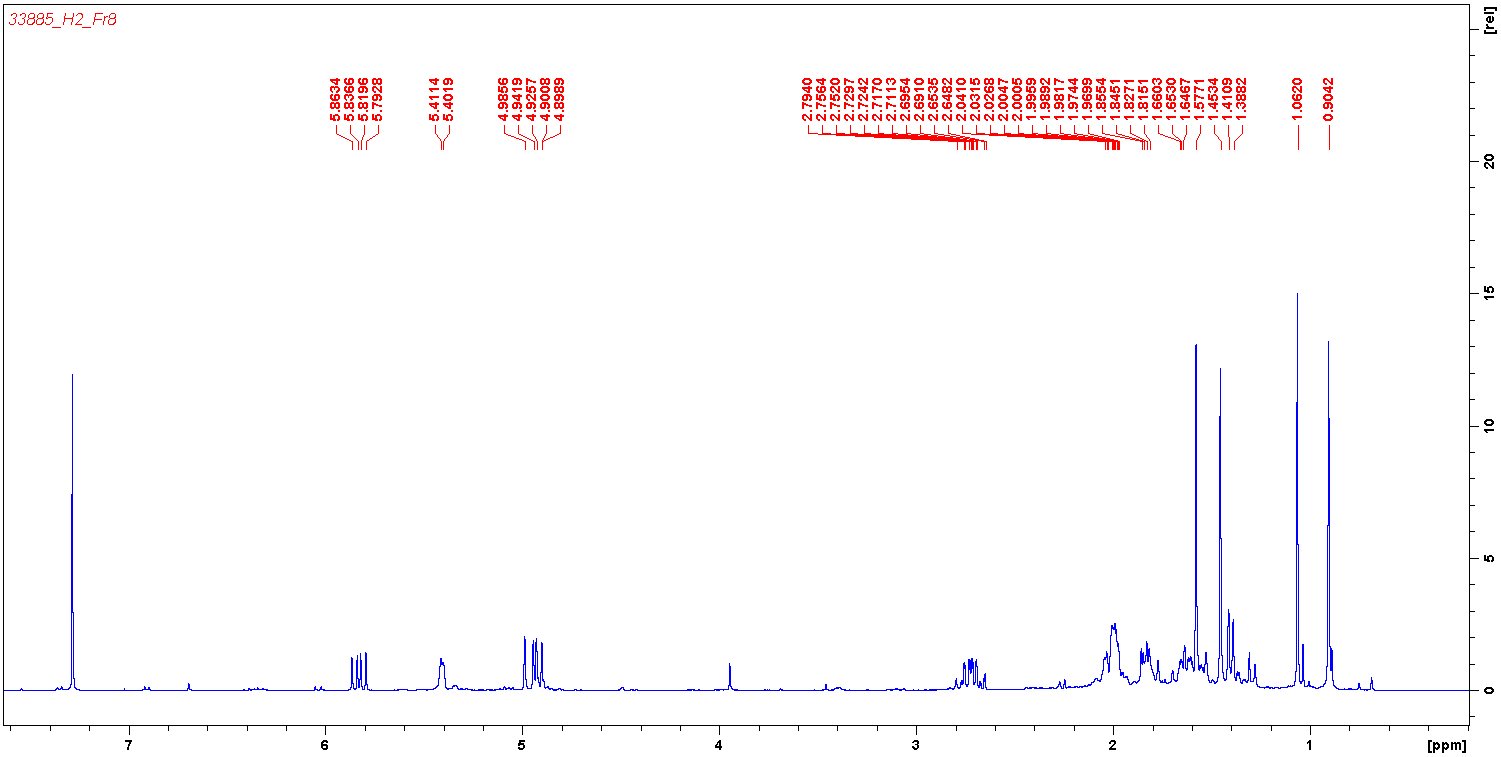


Figure S15. ^1^H NMR spectrum (400 MHz) of compound **3** in CDCl_3_


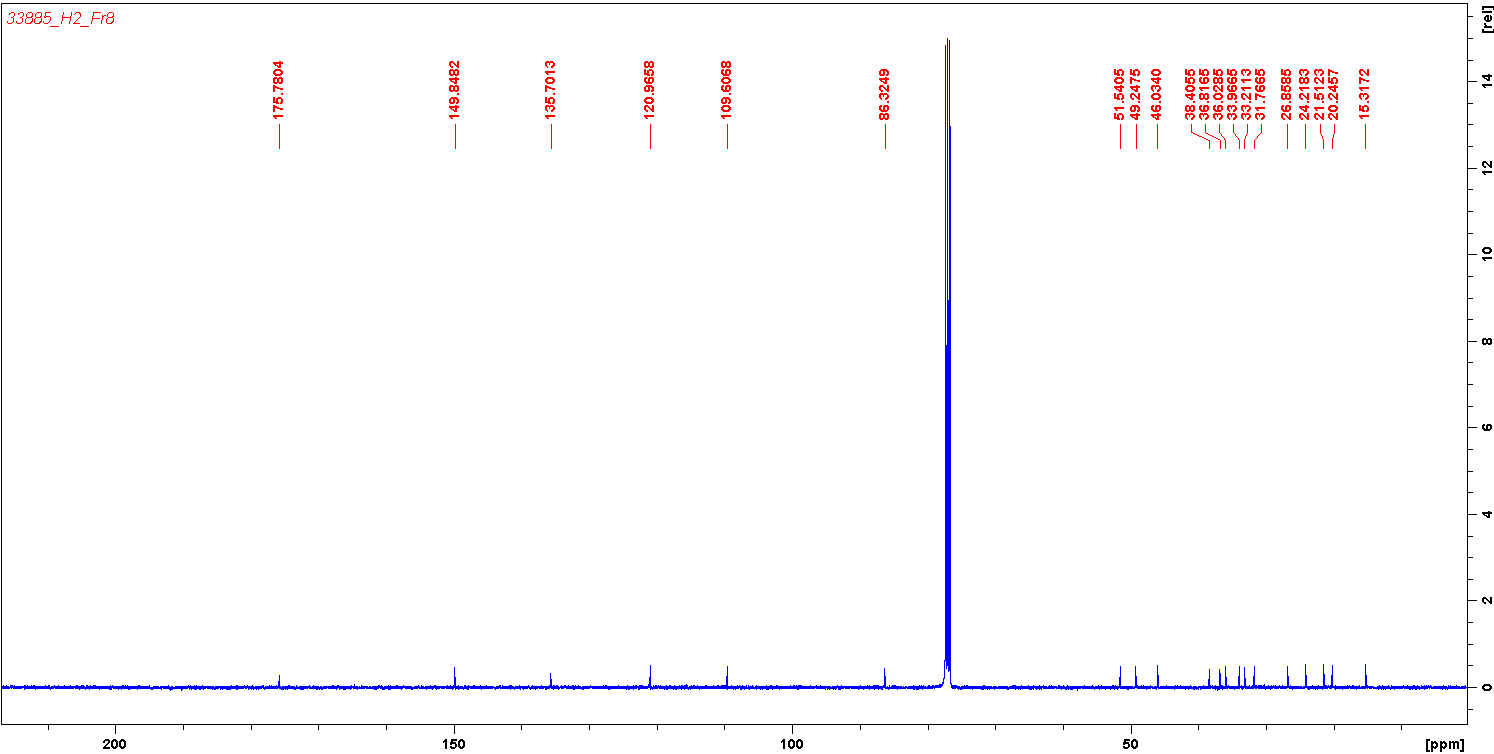


Figure S16. ^13^C NMR spectrum (100 MHz) of compound **3** in CDCl_3_


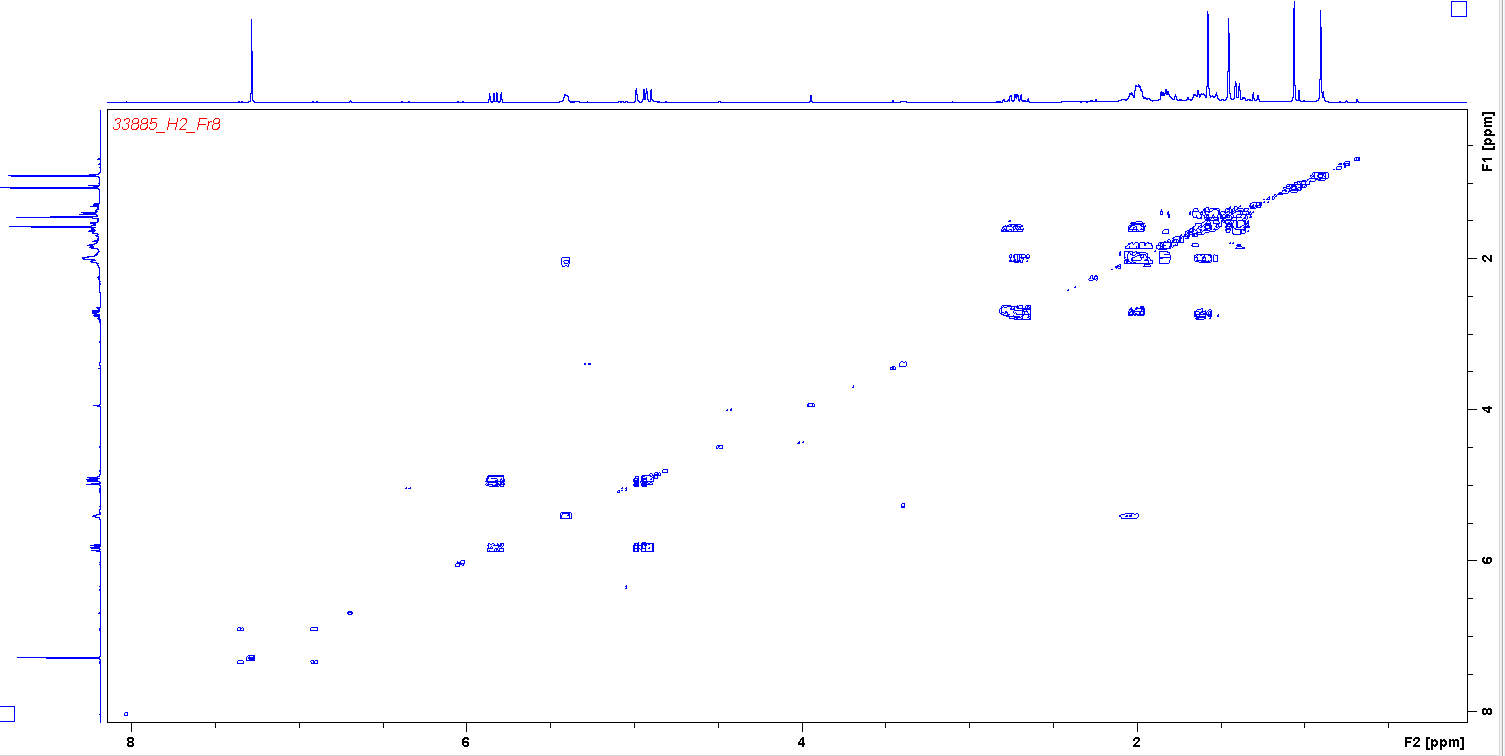


Figure S17. ^1^H,^1^H COSY spectrum of compound **3** in CDCl_3_


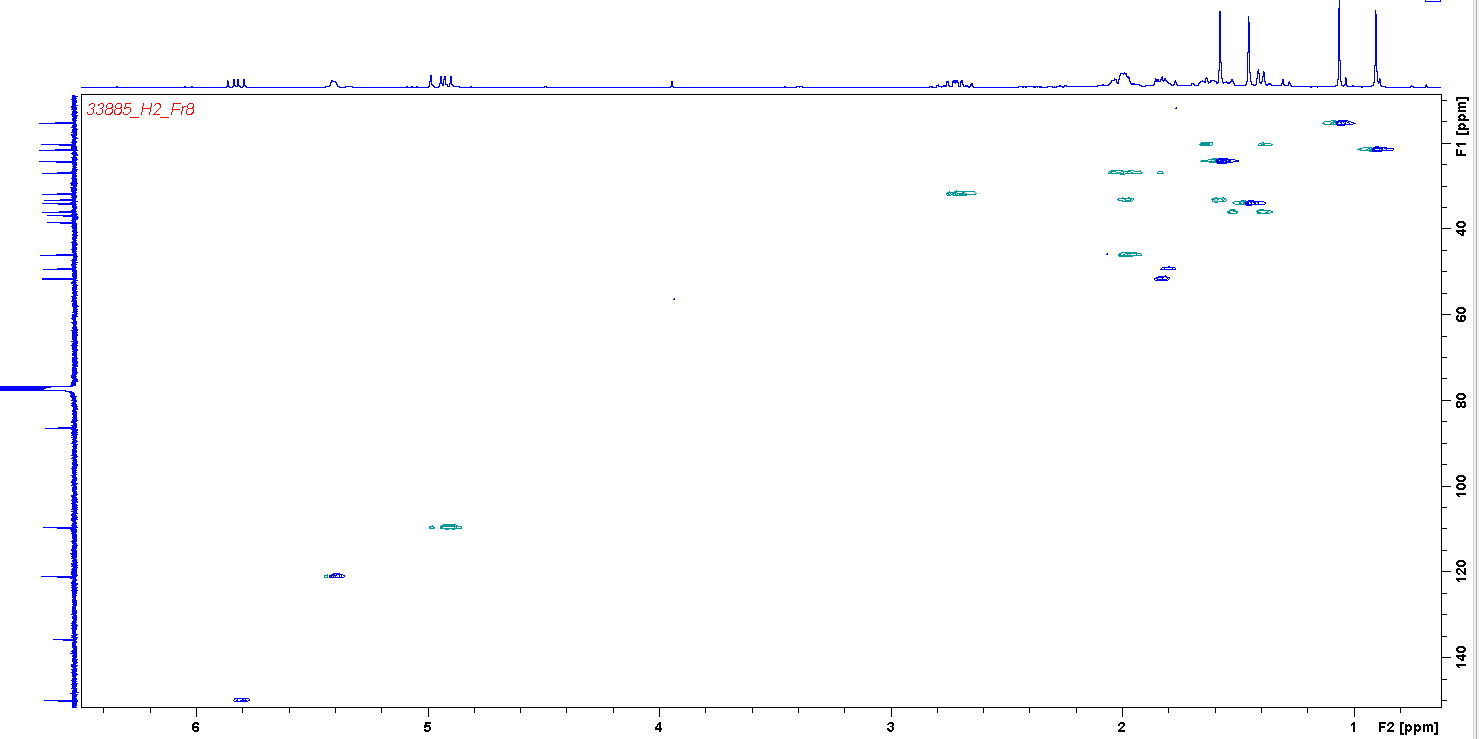


Figure S18. HSQC spectrum of compound **3** in CDCl_3_


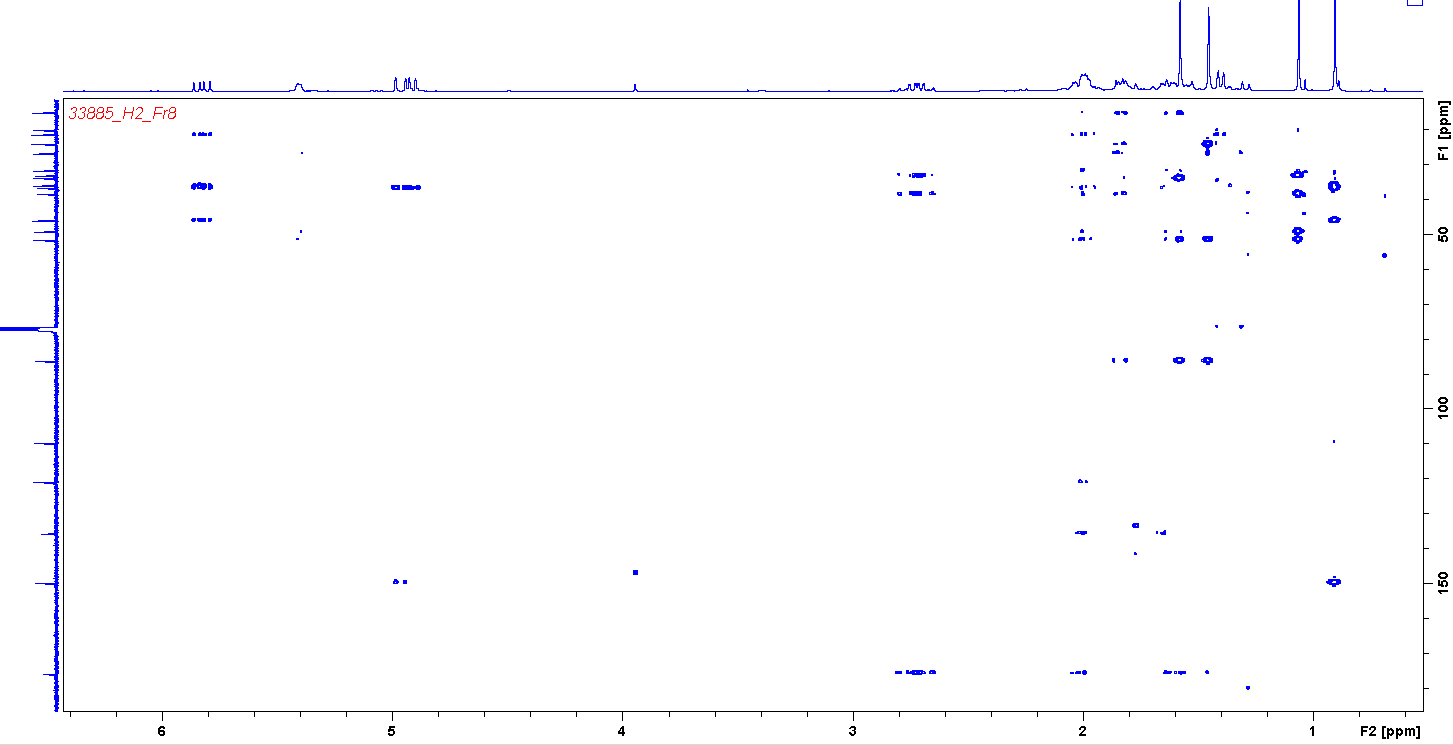


Figure S19. HMBC spectrum of compound **3** in CDCl_3_


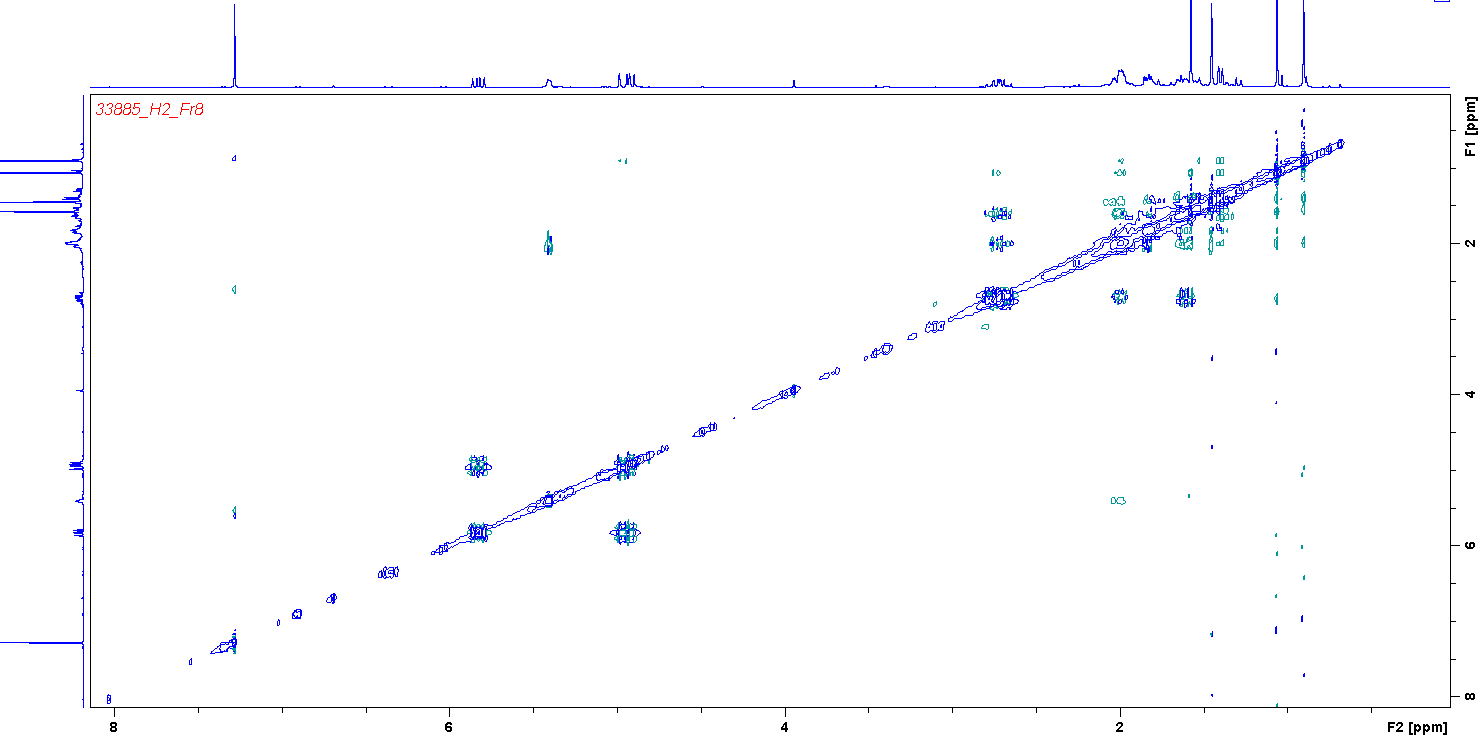


Figure S20. NOESY spectrum of compound **3** in CDCl_3_


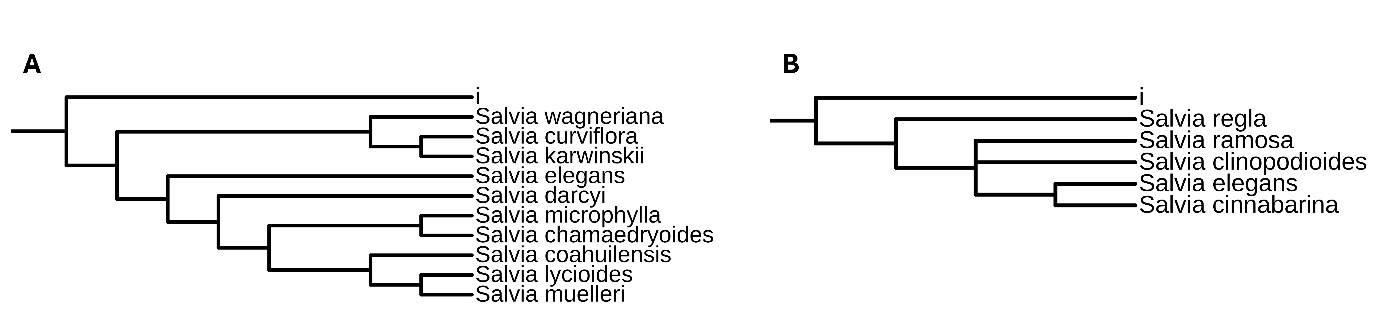


Figure S21. Related species of *S. elegans* as reported by Rose et al.[1] (A) and Lara-Cabrera et al.[2] (B)

Figure S22. Distribution chart of compounds **1**-**3** across *S. elegans* clade

1. Rose JP, Kriebel R, Kahan L, DiNicola A, González-Gallegos JG, et al. Sage Insights Into the Phylogeny of *Salvia*: Dealing With Sources of Discordance Within and Across Genomes. Front. Plant Sci. 2021;12:767478
2. Lara-Cabrera SI, Perez-Garcia M de la L, Maya-Lastra CA, Montero-Castro JC, Godden GT, et al. Phylogenomics of *Salvia* L. subgenus Calosphace

(Lamiaceae). Front. Plant Sci. 2021;12:725900.
